# Inequity of access to contrast-enhanced cardiovascular magnetic resonance in patients with chronic kidney disease: A survey from the British Society of Cardiovascular Magnetic Resonance

**DOI:** 10.1016/j.jocmr.2025.101846

**Published:** 2025-01-26

**Authors:** William E. Moody, Ayisha Mehtab Khan-Kheil, Tamara Naneishvili, Lucy E. Hudsmith, Gabriella Captur, Thomas A. Treibel, Daniel Sado, Timothy Fairbairn, Gerry P. McCann, Saul G. Myerson, Colin Berry, Mark Westwood, Niall G. Keenan

**Affiliations:** a Institute of Cardiovascular Sciences, College of Medical and Dental Sciences, University of Birmingham, Edgbaston, UK; b Department of Cardiology, Queen Elizabeth Hospital Birmingham, University Hospitals Birmingham NHS Foundation Trust, Edgbaston, UK; c The Wolverhampton Heart and Lung Centre, New Cross Hospital, Wolverhampton, UK; d Royal Free London NHS Foundation Trust, London, UK; e Institute of Cardiovascular Science, University College London, London, UK; f Barts Heart Centre, St Bartholomew's Hospital, London, UK; g King’s College Hospital, London, UK; h Liverpool Heart and Chest Hospital, Liverpool, UK; i Department of Cardiovascular Sciences, University of Leicester and the NIHR Leicester Biomedical Research Centre, Glenfield Hospital, Leicester, UK; j Division of Cardiovascular Medicine, Radcliffe Department of Medicine, University of Oxford, Oxford, UK; k School of Cardiovascular and Metabolic Health, BHF Glasgow Cardiovascular Research Centre, Glasgow, UK; l West Herts Teaching Hospitals NHS Trust, Watford, UK; m Institute of Clinical Sciences, Imperial College, London, UK

**Keywords:** Gadolinium-based contrast agent, Chronic kidney disease, Cardiovascular magnetic resonance, Nephrogenic systemic fibrosis

## Abstract

**Objectives:**

To examine the provision of cardiovascular magnetic resonance (CMR) using gadolinium-based contrast agents (GBCA) in patients with chronic kidney disease (CKD).

**Methods:**

An electronic survey was sent to the service leads of all CMR units in the UK in October 2022 requesting information on current departmental protocols and practices.

**Results:**

A response rate of 55% was achieved from the 82 UK CMR units surveyed. There were no known cases of nephrogenic systemic fibrosis (NSF) reported within the past 10 years. Just under half the centers (22 out of 45, 49%) routinely require an estimated glomerular filtration rate (eGFR) in patients before performing contrast-enhanced CMR. Conversely, 18% (8/45) of units do not check eGFR, 20% (9/45) only require an eGFR in patients aged >65 years, while 33% (15/45) assess eGFR in patients known to have CKD. All centers use group II GBCAs: the majority (36/45, 80%) favoring gadobutrol (Gadovist), while gadoterate meglumine (Dotarem) is used in most of the remaining units (8/45, 18%). One in five centers (9/45, 20%) do not currently offer contrast-enhanced CMR to patients with an eGFR <30 mL/min/1.73 m^2^. Of the CMR units that do offer contrast to this group of patients, 28% (10/36) do not obtain consent for the risk of NSF.

**Conclusion:**

One in five centers across the UK does not offer contrast-enhanced CMR to patients with stage 4 and 5 CKD. This finding serves as a call for updated guidance with the intention of standardizing care.

## Graphical abstract

**Figure:**
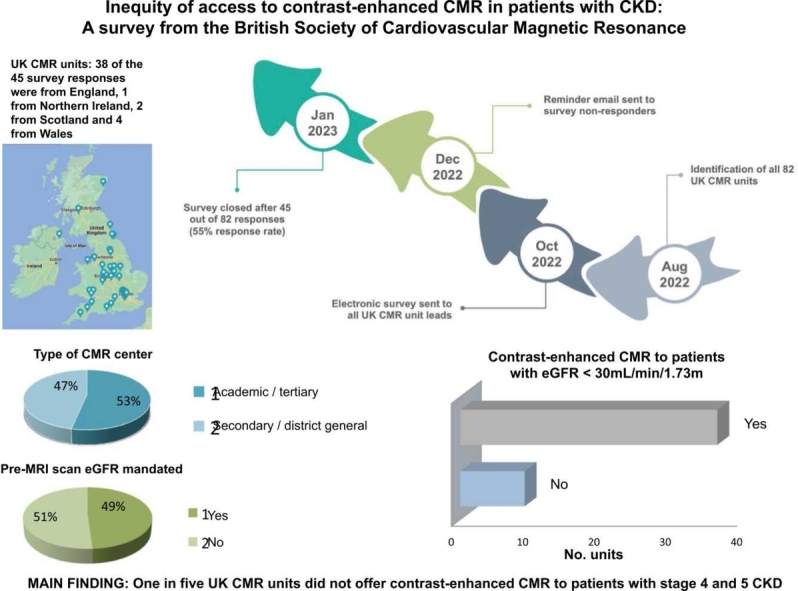

## 1 Introduction

Nephrogenic systemic fibrosis (NSF) is a very rare progressive, multi-organ, fibrosing condition caused by exposure to gadolinium-based contrast agents (GBCAs) used in magnetic resonance imaging (MRI) [1]. It has only been reported in patients with acute kidney injury or stages 4 and 5 chronic kidney disease (CKD) [2]. The majority of patients with the condition suffer debilitating symptoms resulting from organ or skin fibrosis, but it is potentially fatal in a few patients, and there is no curative treatment. After more than 500 cases were reported from 1997 to 2007, the US Food and Drug Administration mandated a black box warning advising avoidance of all GBCAs in at-risk patients [3]. As a result, for several years, many patients with stage 4 or 5 CKD (defined as an estimated glomerular filtration rate [eGFR] <30 mL/min/1.73 m^2^) were denied contrast-enhanced MRI scans, including contrast-enhanced cardiovascular magnetic resonance (CMR). This was despite the reported few hundred cases occurring in the context of hundreds of millions of doses of gadolinium administered during the same period.

Increasing data have emerged that support the concept that not all GBCAs carry the same risk of NSF. In a 2018 recommendation, the American College of Radiology (ACR) emphasized that the lowest-risk GBCAs (so-called “group II” agents, such as gadobenate dimeglumine, gadoteridol, gadoterate meglumine, and gadobutrol), had “very low, if any, risk of NSF” [4]. The ACR guideline also suggested that for group II GBCAs, measurement of eGFR should not be mandated and that contrast-enhanced CMR with a low-risk GBCA should not be denied based on NSF risk. The data supporting this consensus statement have been further strengthened following the findings of a systematic meta-analysis which included 4391 exposures in patients with stages 4 and 5 CKD [5]. Across 16 studies, the pooled incidence of NSF after administration of a group II GBCAs was 0% and the upper bound of the 95% confidence interval was 0.07%. The authors concluded the potential diagnostic harms of withholding group II GBCAs in this population likely outweigh the risk of NSF.

The British Society of CMR (BSCMR; www.bscmr.org) has periodically surveyed CMR units in the UK. Three previous surveys were published in 2011 (2008–2010 data) [6], 2014 (2013 data; abstract only) [7], and 2021 (2017–2018 data) [8] with the principal aim of examining the growth of CMR across the UK. Following the last BSCMR survey, in 2019 the Royal College of Radiologists (RCR) published its own recommendations on GBCA administration [9]. The RCR committee paper included guidance on consent and clarified its recommendation to *not* contraindicate the use of GBCAs in patients with renal impairment. The current BSCMR survey sought to examine: 1) whether UK units are offering GBCAs to patients with stages 4 and 5 CKD undergoing CMR, and 2) if there is regional variation across the UK in the consent process for contrast-enhanced CMR.

## 2 Methods

All CMR units in the UK were identified as described previously [8]. In brief, in England, all 223 National Health Service Trusts/hospitals were reviewed to assess the presence of a CMR unit. In cases of uncertainty, focused enquiries were made by telephoning the cardiology and radiology departments. In Scotland, Northern Ireland, and Wales, where services are centralized, direct approaches were made to MRI departments. For all CMR units, the service lead was identified and sent an electronic survey in October 2022 (full list of questions available in Box 1). In cases of non-response, reminders were sent with follow-up emails. The survey was closed in January 2023.

Box 1 **BSCMR 2022/23 survey questions.**

1. Name of Cardiac MRI center. *Free text*
2. Does your department routinely require an eGFR prior to performing contrast-enhanced CMR (please tick all that apply): *In all patients? / In patients aged over 65 years? / In patients with known chronic kidney disease? / We do not routinely check eGFR.*
3. Have you had any cases of nephrogenic systemic fibrosis in your patient population in the last 10 years? *Yes / No*
4. If your answer to Q3 is yes, survey then asks “How many cases and after what contrast agent?” *Free text*
5. What contrast agent do you use? *Gadobutrol (Gadovist® Gadavist®) / Gadoterate meglumine (Dotarem®) / Free text*
6. Do you offer contrast-enhanced CMR to patients with an eGFR less than 30 mL/min/1.73 m^2^ ? *Yes / No*
7. Do you consent your patients for the risk of nephrogenic systemic fibrosis? *Yes / No*
8. If your answer to Q7 is yes, what process of consent do you use? *Verbal / Written*

## 3 Results

**Fig. 1:**
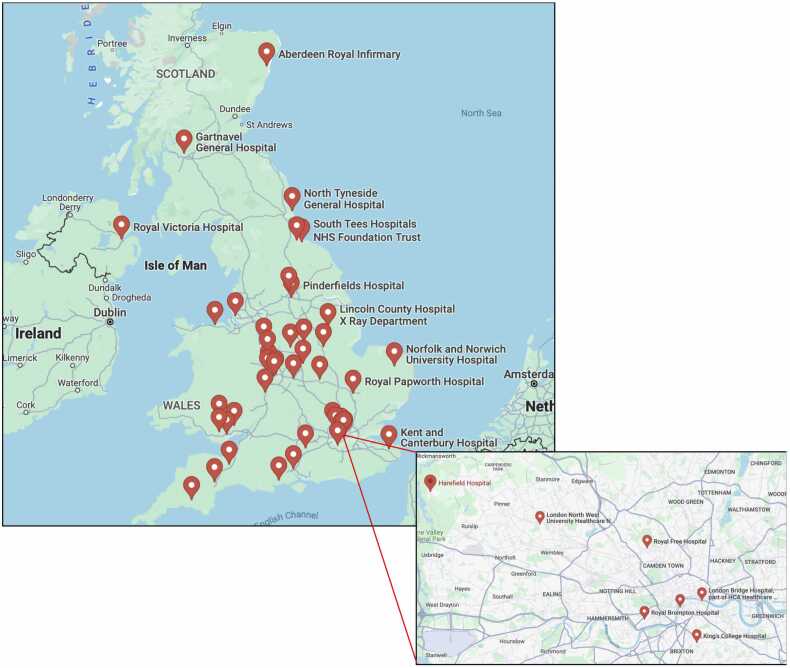
Map showing the locations of the 45 CMR units that responded to the survey. *CMR* cardiovascular magnetic resonance

Of the 82 CMR units identified in the UK, we achieved 45 responses to the survey (55% response rate). Of these, 38 were from England, 1 from Northern Ireland, 2 from Scotland, and 4 from Wales (Fig. 1). University-linked regional centers (tertiary care) accounted for just over half of the respondents (53%, 24 out of 45) with the remainder of survey returns from district general hospitals (secondary care). Approximately half the centers (49%, 22 out of 45) reported requiring an eGFR measurement in *all* patients before giving GBCA while in contrast, 18% (8 out of 45) of centers *do not* routinely check eGFR. A third of centers (15 out of 45, 33%) assess eGFR in patients with known CKD and 20% of units (9 out of 45) require an eGFR in those patients over 65 years of age (figures are not mutually exclusive). The majority of centers (80%, 36 out of 45) use gadobutrol (Gadovist, Bayer Healthcare, Berlin, Germany) as their preferred contrast agent, while gadoterate meglumine (Dotarem [Guerbet, Villepinte, France] or Clariscan [GE Healthcare, Chalfont Saint Giles, UK]) is used in eight centers (18%) and one center (2%) did not specify its choice of GBCA. Notably, 20% (9 out of 45) of centers reported not currently offering contrast-enhanced CMR to any patients with an eGFR less than 30 mL/min/1.73 m^2^. Two out of the nine centers (22%) that do not offer contrast-enhanced CMR to patients with stages 4 and 5 CKD are academic units. Of the 36 CMR units that do offer contrast to this group of patients, 72% (26 out of 36) obtain specific consent for the risk of NSF; of those, 52% (14 out of 26) employ written consent and 57% take verbal consent (15 out of 26, not mutually exclusive). There were no reported cases of NSF in the past 10 years in any of the centers.

## 4 Discussion

The current survey provides contemporary insight into the delivery of contrast-enhanced CMR in patients with CKD across the UK. The main findings are as follows: 1) one in five UK CMR units that responded do not offer contrast-enhanced CMR to patients with stage 4 or 5 CKD, 2) the method of screening for CKD to identify patients at risk of NSF is highly variable across the UK, 3) the process of consenting patients for the risk of NSF associated with GBCA use during CMR examinations differs between centers, and 4) this wide variation in practice exists despite there being no variation in the use of group II GBCAs. The lack of any reported cases from UK CMR centers over the past decade in the current survey is in keeping with the published literature that the risk of NSF from group II GBCM in patients with advanced kidney disease is extremely low (no events following 4931 administrations to patients with stage 4 or 5 CKD) [5].

While we did not specifically ask for the rationale behind the decision for some UK centers to deny patients with CKD stages 4 and 5 access to contrast-enhanced CMR studies, it likely stems from historical concerns regarding the risk of NSF, despite the updated guidance. Based on the current survey data, most (if not all) UK centers currently use group II GBCAs which have the lowest risk of NSF [10]. There are few, if any, cases of NSF that have been associated with group II GBCAs without confounding factors [11], [12], [13]. It was the emergence of these data, which prompted revised guidance in an updated 2021 joint consensus statement by the ACR and the National Kidney Foundation (NKF) [14]. This latest guideline highlights a potential for causing harm by withholding group II GBCA for a clinically indicated MRI in a patient with eGFR <30 mL/min/1.73 m^2^, which could result in delayed diagnosis or misdiagnosis. Emphasizing that no form of dialysis is considered prophylactic for NSF [15], the ACR-NKF statement also differs from the RCR guidance by suggesting that it is not necessary to initiate or alter an established dialysis schedule based on group II GBCA administration [9], [14]. Acknowledging that the dose-related risk of NSF from group II GBCAs is unknown, both guidelines do, however, agree that the lowest diagnostic dose of GBCA (0.1 mmol/kg) should be used [9], [14], avoiding sequential dosing within 7 days in patients with eGFR less than 30 mL/min/1.73 m^2^ [15].

Screening for CKD in patients over the age of 65 years to identify patients at risk for NSF is still supported by the RCR 2019 guidelines [9], but is no longer supported in the ACR-NKF 2021 statement [14]. In line with this, the European Society of Urogenital Radiology and Canadian Association of Radiology have also issued recommendations relaxing the criteria for administration of group II GBCAs in high-risk patients and consider it optional to perform eGFR testing prior to use of group II GBCAs [15], [16]. These discrepancies in the guidelines could explain some of the current variation in UK practice, which ranges from 18% of CMR units having no requirement for eGFR in *any* patient before administration of GBCA, to nearly half the centers (49%) requiring an eGFR in *all* patients. Similarly, the process of consent before the administration of GBCAs across the UK is highly variable. The 2019 RCR guidelines do not stipulate whether written or verbal consent is required but highlight that it is the responsibility of the individual administering the contrast (rather than the referring physician) “to ensure that the patient understands that it is to be given and agrees to proceed” [9]. By contrast, the latest ACR-NKF statement suggests that “informed consent is *not* recommended prior to injection of group II GBCA” [14].

## 5 Limitations

Our survey was limited by its 55% response rate and although it was deliberately focused, we only sourced information regarding the provision of contrast-enhanced CMR in the UK. We do not have data on the demographics of the UK population being served or the indications for CMR, although BSCMR 2021 survey data are available that address this subject [8]. There was an over-representation of London centers in response to the current survey, although this partly reflects the concentration of CMR centers in the capital city [8]. Nonetheless, these findings may have wider implications for the delivery of all contrast-enhanced MRI studies in patients with CKD across the UK.

## 6 Conclusions

There is considerable variation in practice around the UK regarding the testing for renal disease in patients undergoing contrast-enhanced CMR: 20% of UK CMR units deny access to contrast-enhanced CMR for patients with CKD stages 4 and 5 unnecessarily, with implications for their care. Updated guidance from national societies is needed to help improve the standardization of patient care.

## Author contributions

Colin Berry: Writing—review and editing, Supervision. Mark Westwood: Writing—review and editing, Supervision, Resources, Methodology, Conceptualization. William E. Moody: Writing—review and editing, Writing—original draft, Supervision, Formal analysis, Data curation, Conceptualization. Gerry P. McCann: Writing—original draft, Methodology, Conceptualization. Saul G. Myerson: Writing—review and editing, Validation, Methodology, Conceptualization. Lucy E. Hudsmith: Writing—review and editing, Methodology, Conceptualization. Gabriella Captur: Writing—review and editing, Methodology, Conceptualization. Niall G. Keenan: Writing—review and editing, Supervision, Resources, Conceptualization. Ayisha Mehtab Khan-Kheil: Writing—original draft, Data curation. Tamara Naneishvili: Writing—original draft, Project administration, Conceptualization. Timothy Fairbairn: Writing—original draft, Methodology, Conceptualization. Thomas A. Treibel: Writing—review and editing, Methodology, Conceptualization. Daniel Sado: Writing—review and editing, Methodology, Conceptualization.

## Declaration of competing interests

None.
